# Supplementary material for: An NKX2-1/ERK/WNT feedback loop modulates gastric identity and response to targeted therapy in lung adenocarcinoma
Source: eLife. 2021 Apr 6;10:e66788. doi: 10.7554/eLife.66788 (PMC8102067; doi:10.7554/eLife.66788)
Supplement: Transparent reporting form [file elife-66788-transrepform.docx]

***eLife’s* transparent reporting form**

We encourage authors to provide detailed information *within their submission* to facilitate the interpretation and replication of experiments. Authors can upload supporting documentation to indicate the use of appropriate reporting guidelines for health-related research (see [EQUATOR Network](http://www.equator-network.org/%20)), life science research (see the [BioSharing Information Resource](https://biosharing.org/)), or the [ARRIVE guidelines](http://www.plosbiology.org/article/info:doi/10.1371/journal.pbio.1000412) for reporting work involving animal research. Where applicable, authors should refer to any relevant reporting standards documents in this form.

If you have any questions, please consult our Journal Policies and/or contact us: [editorial@elifesciences.org](mailto:editorial@elifesciences.org).

**Sample-size estimation**

- You should state whether an appropriate sample size was computed when the study was being designed
- You should state the statistical method of sample size computation and any required assumptions
- If no explicit power analysis was used, you should describe how you decided what sample (replicate) size (number) to use

Please outline where this information can be found within the submission (e.g., sections or figure legends), or explain why this information doesn’t apply to your submission:

No sample-size calculation was performed. However, we used scRNA-seq in this study, which generates thousands of cells per sample. And all main conclusions in the study were corroborated by multiple approaches both in vivo and ex vivo.

Sample sizes for in vitro and mouse experiments were determined according to the minimal number of independent biological replicates that significantly identified an effect, incorporating previous experience with degree of variability in model systems. Experiments were performed at least in triplicate independent measurements or more as indicated in Figure legends and main text.

**Replicates**

- You should report how often each experiment was performed
- You should include a definition of biological versus technical replication
- The data obtained should be provided and sufficient information should be provided to indicate the number of independent biological and/or technical replicates
- If you encountered any outliers, you should describe how these were handled
- Criteria for exclusion/inclusion of data should be clearly stated
- High-throughput sequence data should be uploaded before submission, with a private link for reviewers provided (these are available from both GEO and ArrayExpress)

Please outline where this information can be found within the submission (e.g., sections or figure legends), or explain why this information doesn’t apply to your submission:

**Replication**: Most experiments in this study were independently replicated, with biological and technical replicates listed in the legends of the corresponding figures with similar results. RNA sequencing experiments (bulk and single cell) were done once but multiple biological replicates were included in each experiment. Findings from bulk RNAseq and scRNA-seq, done independently and from different mice, were similar.

**Data exclusion**: In our bulk RNAseq, we determined that one sample (id 14489X13) did not group with other samples in the MultiQC report. it was later confirmed that very limited starting material and poor RNA quality were contributing factors to its difference from the others. This sample was removed from downstream analysis because the noise in the sample was likely a larger trade-off than the increase in biological replicates for that condition.

In scRNAseq, cell selection and filtering are detailed in the Methods section.

**Statistical reporting**

- Statistical analysis methods should be described and justified
- Raw data should be presented in figures whenever informative to do so (typically when N per group is less than 10)
- For each experiment, you should identify the statistical tests used, exact values of N, definitions of center, methods of multiple test correction, and dispersion and precision measures (e.g., mean, median, SD, SEM, confidence intervals; and, for the major substantive results, a measure of effect size (e.g., Pearson's r, Cohen's d)
- Report exact p-values wherever possible alongside the summary statistics and 95% confidence intervals. These should be reported for all key questions and not only when the p-value is less than 0.05.

Please outline where this information can be found within the submission (e.g., sections or figure legends), or explain why this information doesn’t apply to your submission:

Raw data are shown as dot plots where appropriate. Statistical test methods and p-values for each panel can be found in figure legends and are detailed in Methods section.

(For large datasets, or papers with a very large number of statistical tests, you may upload a single table file with tests, Ns, etc., with reference to sections in the manuscript.)

**Group allocation**

- Indicate how samples were allocated into experimental groups (in the case of clinical studies, please specify allocation to treatment method); if randomization was used, please also state if restricted randomization was applied
- Indicate if masking was used during group allocation, data collection and/or data analysis

Please outline where this information can be found within the submission (e.g., sections or figure legends), or explain why this information doesn’t apply to your submission:

**Randomization**: In mouse drug-treatment experiments, mice were randomly assigned to each experimental group.

**Blinding**: In most cases, investigators were not blinded to allocation during experiments and outcome assessment. Blinding was not possible as the same investigator processed the organoids/animals and analyzed the data.

However, RNA sequencing was generated by one investigator and analyses were performed by two independent investigators who were blinded to the experimental design and sample genotype at the first stages of analyses.

Histopathologic analysis was generally not blinded because significant morphologic differences between genotypes/treatments made the experimental group obvious. However, for Figure 8C, IHC slides were blinded by one investigator and MCM2 quantitations performed by another investigator.

**Additional data files (“source data”)**

- We encourage you to upload relevant additional data files, such as numerical data that are represented as a graph in a figure, or as a summary table
- Where provided, these should be in the most useful format, and they can be uploaded as “Source data” files linked to a main figure or table
- Include model definition files including the full list of parameters used
- Include code used for data analysis (e.g., R, MatLab)
- Avoid stating that data files are “available upon request”

Please indicate the figures or tables for which source data files have been provided:

Complete list of DEG bulk RNAseq and scRNA-seq are provided in Supplementary Files 1-4.

GO analysis data on bulk RNAseq from Illumina Correlation Engine (for Figures 5C and 5D) are provided in Supplementary Files 5 & 6.

Complete RNA sequencing data have been deposited in the Gene Expression Omnibus (GEO, NCBI) repository under accession number GSE145152, with a reviewer token utulgaumnvafxob to access the data.

No restrictions on data availability.
